# Single Nucleotide Polymorphism in the *ADIPOQ* Gene Modifies Adiponectin Levels and Glycemic Control in Type Two Diabetes Mellitus Patients

**DOI:** 10.1155/2022/6632442

**Published:** 2022-04-27

**Authors:** Mahmoud A. Alfaqih, Aisha Al-hawamdeh, Zouhair O. Amarin, Yousef S. Khader, Khawla Mhedat, Mohammed Z. Allouh

**Affiliations:** ^1^Department of Physiology and Biochemistry, Faculty of Medicine, Jordan University of Science and Technology, Irbid 22110, Jordan; ^2^Department of Obstetrics and Gynecology, Faculty of Medicine, Jordan University of Science and Technology, Irbid 22110, Jordan; ^3^Department of Public Health and Community Medicine, Faculty of Medicine, Jordan University of Science and Technology, Irbid 22110, Jordan; ^4^Department of Anatomy, College of Medicine and Health Sciences, United Arab Emirates University, Al-Ain 15551, UAE

## Abstract

Diabetes mellitus (DM) is the ninth leading cause of death worldwide. Mortality from DM is largely attributed to disease complications. Glycemic control of DM patients reduces mortality. Studies indicated that the lack of glycemic control in DM patients could be influenced by the genetic background of the patients. Evidence suggests that adiponectin levels are dysregulated in DM patients with poor glycemic control. Serum adiponectin level is a heritable trait influenced by single nucleotide polymorphisms (SNPs) in the *ADIPOQ* gene. It is hypothesized that SNPs in *ADIPOQ* could modify glycemic control in DM patients. To test this hypothesis, 375 type 2 DM (T2DM) patients were recruited. Patients were classified into good vs. poor glycemic control according to hemoglobin A1c levels. Study subjects were genotyped for variations of four SNPs in *ADIPOQ* (rs17300539, rs266729, rs2241766, and rs1501299). Adiponectin levels were measured from the serum. Our analysis showed that reduced serum adiponectin, a longer duration of treatment, and increased insulin resistance were all significant predictors of poor glycemic control. Moreover, the T allele and the TT genotype of rs2241766 were significantly more frequent in patients with poor glycemic control (*P* < 0.05). Individuals with the TT genotype of rs2241766 had significantly lower levels of serum adiponectin (*P* < 0.05). It was concluded that lower levels of serum adiponectin and the T allele of rs2241766 SNP in *ADIPOQ* were associated with poor glycemic control in T2DM patients.

## 1. Introduction

Diabetes mellitus (DM) is a complex multifactorial disease characterized by chronic hyperglycemia [1]. Type 2 DM (T2DM) represents 90-95% of all DM cases and is caused by a relative deficiency of insulin secretion accompanied by resistance to its activity [1]. T2DM is a global public health problem and was estimated to be linked to 1 out of 9 deaths in individuals in the age range of 20-79 years [2].

The development of DM is associated with a plethora of complications [3]. Most of these complications are life-threatening. The onset of these complications partially explains the high mortality rates associated with DM [3]. For example, DM is associated with an increased risk of hypertension [4], atherosclerosis [5], and renal failure [6]. The International Diabetes Federation (IDF) estimated that DM or its complications are responsible for 5 million deaths in the year 2015 alone [7]. This figure is equivalent to one death every six seconds [7].

A growing body of evidence indicates that most DM complications are either directly or indirectly related to hyperglycemia [8–10]. Accordingly, maintaining blood glucose levels within a standardized reference range remains a major goal of managing DM patients [11]. Several reports support that controlling blood glucose levels in DM patients delays the onset and reduces the severity of complications [12, 13]. According to the most recent guidelines of the American Diabetes Association (ADA), the proper management of T2DM requires maintaining glycosylated hemoglobin levels (hemoglobin A1c (HbA1c)) at or below 7% [14].

Despite the emphasis on controlling blood glucose levels in most DM management protocols, many patients still fail to reach optimum levels of glycemic control [15]. Many factors were found to modify the risk of poor glycemic control among T2DM patients, including age, obesity, duration of DM, and adherence to therapeutic protocols [16–18].

Insulin resistance (IR), a condition in which tissues fail to utilize glucose for energy and metabolism, is a significant contributing factor to poor glycemic control [19]. The development of IR is influenced by obesity [20], physical inactivity [21], and genetic predisposition [22]. Nonetheless, the cause of IR remains multifactorial. Moreover, the exact mechanism that explains the role of IR in the glycemic control of T2DM patients remains a rich area for investigation.

Adiponectin is a protein expressed and secreted by adipose tissues [23]. One of the many metabolic effects attributed to adiponectin activity is its ability to enhance insulin action on its target tissues [24]. Not surprisingly, several observational studies found that the serum level of adiponectin is reduced in chronic diseases where IR is a major predisposing factor. For example, adiponectin expression is downregulated in obesity [25]. Moreover, lower adiponectin levels increase the risk of prediabetes [26], T2DM [27], and polycystic ovarian syndrome [28].

Variations in serum adiponectin levels among individuals could be explained by differences in adipose tissue distribution. However, one genetic study found that 80% of the variance in serum adiponectin level among nonobese subjects could be explained by additive genetic effects [29]. The above observation was further supported by several studies, which found that single nucleotide polymorphisms (SNPs) in the *ADIPOQ* gene (the gene that codes for adiponectin protein) are associated with differences in serum adiponectin [30–32]. In conclusion, it appears that variation in serum adiponectin could be genetically determined.

A literature review highlights several SNPs in the ADIPOQ gene associated with variations in serum adiponectin. Based on our discussion above, any of these SNPs could theoretically modify the risk of poor glycemic control in T2DM patients. Rs17300539 is a SNP located in the promoter region of the ADIPOQ gene and is believed to be involved in regulating ADIPOQ expression [33]. Indeed, a report that included participants from the Framingham Offspring Study (*n* = 2543) found that rs17300539 was strongly associated with serum adiponectin levels in the study subjects [34].

Rs266729 is a SNP of the ADIPOQ gene believed to be involved in regulating promoter activity. In a study that included a population of 1004 adult obese participants, the G allele of rs266729 was found to be associated with lower levels of serum adiponectin and a higher risk of hyperglycemia [35]. Another SNP in the ADIPOQ gene associated with differences in serum adiponectin is rs2241766. In a cross-sectional study of 242 subjects ethnically classified as Mexican-Mestizo, it was demonstrated that individuals who carry the GG genotype of rs2241766 have significantly higher levels of serum adiponectin than individuals who carry the TT or the TG genotypes of the SNP [36]. Lastly, Rs1501299 is an intronic variant of the ADIPOQ gene [28]. Several reports support the association of rs1501299 with variations in serum adiponectin. Indeed, de Luis et al. showed that individuals who carry the T allele of this SNP were at higher risk of developing hyperglycemia and/or the metabolic syndrome [37]. This is presumably due to an association of the T allele with lower serum adiponectin levels [37].

Given that serum adiponectin modifies IR and the role IR plays in controlling glucose levels in T2DM, we tested the association between serum adiponectin and the lack of glycemic control in a population of T2DM patients. Using the same population, we also evaluated the association of four SNPs in the *ADIPOQ* gene (rs2241766, rs1501299, rs266729, and rs17300539) with the lack of glycemic control.

## 2. Methods

### 2.1. Study Design and Subject Recruitment

This case-control study involved 350 T2DM patients of Jordanian descent who presented at the endocrinology and diabetes clinics of King Abdullah University Hospital (KAUH), a tertiary hospital located in the Northern part of Jordan. All patients recruited to the study had a confirmed diagnosis of T2DM according to the guidelines of the American Diabetes Association and were actively treated for their illness at the time of their recruitment. Before starting to recruit patients, the study was approved by KAUH Institutional Review Board (IRB).

Patient recruitment involved a short interview with the patient by a clinical research coordinator. In the interview, the coordinator briefly explained the objectives of the study and that participation in the study would involve collecting demographic and clinical data, obtaining anthropometric measurements, and a future blood withdrawal. If any of the patients agreed to participate in the study, they were then requested to sign an informed consent, which also reiterated the information detailed above.

During the patients' next visit, demographic, anthropometric, and clinical data were collected. Demographic data included age and gender, while anthropometric data included weight (in kilograms (kg)), height (in meters (m)), and waist circumference (WC) (in centimeters (cm)). Clinical data included the specific type of medication the patient was using at the time of recruitment, compliance to diabetes treatment, and the length of time the patient had been diagnosed with T2DM and actively receiving treatment for their condition. Compliance with diabetes treatment was first assessed by directly asking the patients if they regularly took their medication. This was followed by evaluating the patients' medical records to ascertain the presence of regular prescription filling patterns originating from the date the patient started receiving treatment at KAUH. Patients who missed more than three consecutive refills were considered noncompliant and were excluded from the study.

Thiazolidinediones are drugs that primarily function via binding to peroxisome proliferator-activated receptor gamma (PPAR-*γ*) and may modify serum adiponectin levels [38]. Therefore, patients receiving thiazolidinediones were excluded from the study. Insulin is expected to affect HOMA-IR, and thus, patients using insulin of any type were also excluded. Patients that were only receiving metformin were included in the final analysis. Other exclusion criteria included the presence of any reference in the patient's electronic record for the presence of any of the following diabetic complications: retinopathy, neuropathy, nephropathy, and atherosclerosis. The collected demographic, anthropometric, and clinical data were then entered into Excel spreadsheets.

### 2.2. Blood Sample Collection

Two blood samples (7 ml each) were collected from each study subject by a certified phlebotomist. Subjects were requested to fast for 15 hours before blood withdrawal, performed the following morning at around 9 am. For HbA1c measurement and DNA extraction, one blood sample was collected into an ethylene-diamine-tetra-acetic acid (EDTA) tube (AFCO, Jordan) and then kept at 4°C. The other sample was collected into a plain tube with a gel clot activator (AFCO, Jordan) and used to obtain serum following centrifugation for 5 minutes at 4000 × *g*. The supernatant (i.e., serum) was then transferred into an Eppendorf tube and stored at -80°C.

### 2.3. HbA1c Measurement

A fraction of blood (around 5 ml) stored in EDTA tubes was used to measure HbA1c levels. These samples were submitted to the laboratories of KAUH, and measurements were performed using an automated analyzer system (Roche Diagnostics, Mannheim, Germany). HbA1c measurements were then used to gauge the glycemic control of the patients. Specifically, if HbA1c levels were lower than 7%, the individual was considered to have good glycemic control. On the other hand, if HbA1c levels were higher than or equal to 7%, the individual was considered to have poor glycemic control. We recruited a total of 175 T2DM patients of good glycemic control and another 175 patients of poor glycemic control. The two groups were matched by age, BMI, and type of diabetes treatment.

### 2.4. Biochemical Measurements

The serum stored at -80°C was later used to measure glucose, total cholesterol, triglycerides, and adiponectin. Measurements of serum glucose, total cholesterol, and triglyceride were performed at the laboratories of KAUH. An enzyme-linked immunosorbent assay (ELISA) was applied to measure serum adiponectin. The ELISA kit used to measure adiponectin was purchased from R&D Systems (Minneapolis, MN), and the measuring protocol was performed according to the method described by Alfaqih et al. [26, 28, 39].

### 2.5. DNA Extraction and Genotyping

Genomic DNA was extracted from whole blood samples collected in EDTA tubes. QIAamp DNA Blood Mini Kit (Qiagen, Hilden, Germany) was used in the procedure. DNA purity was then checked using an ND-2000 NanoDrop (Thermo Scientific, Waltham, MA, USA). A polymerase chain reaction-restriction fragment length polymorphism- (PCR-RFLP-) based approach was used to determine the genotypes of four SNPs of the *ADIPOQ* gene (rs17300539, rs266729, rs1501299, and rs2241766). The concentrations of the reagents used in the PCR reaction and the final reaction volume were as described by Alfaqih et al. [26]. The forward and reverse primer sequence of each SNP can be found in Table 1. The location of each SNP on the *ADIPOQ* gene, the size of the PCR amplicon, the restriction enzyme used in the assay, and the size of the products following restriction enzyme digestion are listed in Table 1. The undigested PCR products and the DNA fragments which resulted from restriction enzyme treatment were run on a 3% agarose gel stained with SYBR™ Safe DNA Gel Stain (ThermoFisher Scientific, Waltham, MA, USA) and then visualized under blue light.

### 2.6. Statistical Analysis

Statistical analysis was performed using the Statistical Package for Social Studies (SPSS) software (version 23, IBM, NY). Differences in age, BMI, WC, total cholesterol, triglyceride, treatment duration, HbA1c, glucose, HOMA-IR, and adiponectin levels between poorly controlled patients and patients of good glycemic control were evaluated using the Student's *t*-test. Differences in gender distribution between the two above groups were assessed using Pearson's chi-square test of association. The association between allele or genotype categories with the risk of poor glycemic control was also assessed using Pearson's chi-square test. Differences in serum adiponectin levels between the different genotype categories of rs2241766 were evaluated using the one-way ANOVA test followed by Tukey's post hoc analysis. SHEsis software was used to run haplotype analysis [40]. Multivariate regression analysis included the following variables: serum adiponectin, treatment duration, cholesterol, triglyceride, and HOMA-IR. A *P* value of 0.05 and a 95% confidence interval were considered statistically significant.

## 3. Results

### 3.1. Serum Adiponectin Level is Lower in T2DM Patients with Poor Glycemic Control

Baseline characteristics of the study subjects are shown in Table 2. Our analysis showed that T2DM patients with poor glycemic control were actively treated for their illness for a significantly longer duration of time than patients with good glycemic control (*P* < 0.0001). Furthermore, these patients had significantly higher HbA1c and fasting serum glucose levels (*P* < 0.05). Patients with poor glycemic control also had a significantly higher value of HOMA-IR index (*P* < 0.0001) but a significantly lower level of serum adiponectin relative to patients with good glycemic control (*P* < 0.0001).

Differences in HOMA-IR index could explain lower levels of serum adiponectin in T2DM patients with poor glycemic control. Therefore, we tested the association of serum adiponectin levels with poor glycemic control using multivariate regression. It was observed using this analysis that serum adiponectin remained significantly associated with poor glycemic control and reduced its risk (OR 0.974; CI 0.949-0.999; *P* = 0.043) (Table 3). In the above model, treatment duration and HOMA-IR were also significant independent predictors of poor glycemic control.

### 3.2. Association of rs2241766 in *ADIPOQ* with Poor Glycemic Control

The association of several SNPs in the *ADIPOQ* gene with the poor glycemic control condition was investigated. We used a PCR-RFLP-based approach to determine the genotype of study subjects for the following SNPs (rs17300539, rs266729, rs2241766, and rs1501299). The results of this analysis are shown in Table 4. Our findings showed that rs2241766 was significantly associated with poor glycemic control (*P* < 0.05).

Our analysis of genotype distribution of rs2241766 determined that the percentage of T2DM patients with the TT genotype of rs2241766 was higher in patients with poor glycemic control (Table 4). On the other hand, the percentage of T2DM patients with the heterozygous TG genotype or the homozygous GG genotype was lower in patients with poor glycemic control.

Along the same lines, it was observed that the percentage of T2DM patients with the G allele of rs2241766 was significantly lower in patients with poor glycemic control (Table 5). We conclude that the minor G allele of rs2241766 could reduce the risk of poor glycemic control among T2DM patients.

The above conclusion was further supported by the findings of our haplotype analysis shown in Table 6. This analysis identified that the frequency of GCGG haplotype of *ADIPOQ*, which contains the minor G allele of rs2241766, was significantly lower among T2DM patients with poor glycemic control (OR 0.500; CI 0.341-0.734; *P* = 0.0003).

### 3.3. Genetic Variation in rs2241766 Affects Serum Adiponectin Levels

**Figure 1:**
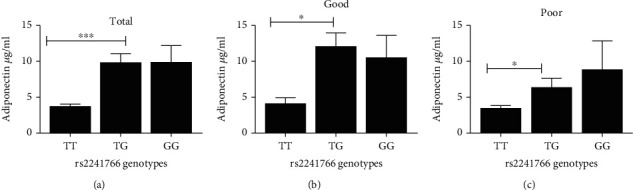
Effect of the genotype class of rs2241766 polymorphism of the ADIPOQ gene on serum adiponectin levels. Study subjects were categorized according to their rs2241766 genotype class (TT, TG, or GG), and the serum levels of adiponectin were compared between the three different classes. This analysis was performed on (a) total study population, and type 2 diabetic patients with (b) good and (c) poor glycemic control. Each column represents the mean ± standard error of the mean. ^∗^*P* < 0.05 and ^∗∗∗^*P* < 0.001. Significant differences between the different genotype classes (ANOVA, Tukey's post hoc).

Given our findings that genetic variation in rs2241766 of *ADIPOQ* gene modified the risk of poor glycemic control, we wanted to test if genetic variations in that locus were associated with differences in serum adiponectin levels in T2DM patients of our population. To achieve this goal, we compared serum adiponectin levels between different genotype categories of rs2241766. We found that serum adiponectin levels were significantly higher in T2DM patients with the TG genotype of rs2241766 than patients with the TT genotype of rs2241766 (Figure 1). Interestingly, serum adiponectin levels in T2DM patients with the GG genotype of rs2241766 were also higher than patients with the TT genotype (Figure 1); however, these differences did not reach statistical significance. Noteworthy, significantly higher serum adiponectin levels in T2DM patients with the TG genotype than the TT genotype were observed in good glycemic control (Figure 1(b)) and poor glycemic control (Figure 1(c)) patients.

## 4. Discussion

The findings of this study add to previously existing data suggesting that adiponectin, one of its metabolites, or an effector downstream of its receptor may modulate the risk of poor glycemic control in T2DM patients. Furthermore, this report also demonstrated that genetic variations in the *ADIPOQ* gene are associated with the risk of poor glycemic control. The above findings will help further our understanding of the pathobiology of glycemic control and its risk and highlight several pharmacological and nonpharmacological approaches that can be used to better manage T2DM. Moreover, the results of this study add to the growing body of evidence that supports the notion that glycemic control in T2DM patients is both genetically and environmentally determined.

A decrease in adiponectin levels was observed in T2DM patients who lack glycemic control. The above association between lower adiponectin and the lack of glycemic control remained significant in our multivariate model following adjustment for treatment duration, serum cholesterol, serum triglyceride, and HOMA-IR. This finding coincides with Schulze et al. [41], who observed that higher plasma adiponectin levels were associated with lower HbA1c levels. However, the above report did not use a case-control design, and T2DM patients were not categorized using ADA guidelines into patients of good vs. poor glycemic control, a design we used in this study. Al-Azzam et al. [42] categorized T2DM subjects into patients with good or poor glycemic control and measured adiponectin levels in serum samples recovered from the study subjects. However, in their report, adiponectin levels were not significantly different between the two categories, although Al-Azzam et al. [42] reported that genetic variation in the *ADIPOQ* gene was associated with the risk of poor glycemic control.

The exact mechanism that explains the positive relationship between higher serum adiponectin and good glycemic control is currently unknown. This relationship could be explained by the fact that adiponectin enhances insulin action on target tissues (i.e., insulin sensitivity). However, in this report, we found in our multivariate model that the association of adiponectin with good glycemic control was independent of HOMA-IR, an index of insulin resistance. This observation favors that other mechanisms could explain the relationship between adiponectin and good glycemic control. For example, adiponectin could be enhancing glycemic control in T2DM patients via directly inhibiting hepatic gluconeogenesis, a well-established effect of adiponectin [43].

Another contemporary opinion could be that higher adiponectin is associated with better glycemic control through an anti-inflammatory effect of adiponectin. Indeed, there is growing evidence that many metabolic disorders are associated with chronic mild inflammation [44], including lack of glycemic control.

A causal relationship between low levels of serum adiponectin and poor glycemic control was not tested per se in this report. However, our findings indicate that increasing serum adiponectin levels may help in the management of poor glycemic control or in mitigating its effects. An increase in serum adiponectin could be achieved via either pharmacological or nonpharmacological means. It is established that the use of agonists of the peroxisome proliferator-activated receptor gamma (PPAR-*γ*) can increase the levels of serum adiponectin [45, 46]. The use of such agonists or functionally similar ligands could be tested for their efficacy in achieving glycemic control in well-designed clinical trials. Foula et al. [47] described an interventional study on 95 obese or overweight premenopausal females enrolled in a controlled weight reduction program based on a balanced low-calorie diet. In their report, Foula et al. [47] observed a significant decrease in the weight of females enrolled in the trial accompanied by a substantial increase in serum adiponectin. Nonpharmacological interventions of a similar design could be tested on T2DM patients with poor glycemic control to ascertain if these interventions could cause an increase in serum adiponectin accompanied by better glycemic control.

In this report, we observed that genetic variation in the *ADIPOQ* gene was associated with lower serum adiponectin levels. Specifically, we found that T2DM patients of the TT genotype of rs2241766 had lower serum adiponectin levels. Interestingly, we also observed that the T allele of rs2241766 was associated with a higher risk of poor glycemic control. These findings indicate that genetic variants which affect serum adiponectin levels may also affect the risk of poor glycemic control. Additionally, these findings demonstrate that serum adiponectin level is a heritable trait that could predispose individuals to certain metabolic disorders.

In this study, the research team assumed that rs2241766 modified the risk of poor glycemic control via a direct effect on serum adiponectin levels. However, this may not be the sole mechanism that explains the above observation. For example, rs2241766 could be in linkage disequilibrium with another close by genetic variant that modifies the expression of a different gene, which belongs to a different signaling pathway unrelated to signaling pathways downstream of adiponectin receptor. The exclusion of such a scenario may require whole-genome association studies with larger sample size and is outside the scope of the current investigation.

Several reports investigated the association of rs2241766 of the *ADIPOQ* gene with several metabolic disorders believed to be predisposed by insulin resistance. For example, to determine an association between rs2241766 and T2DM risk, Dong et al. performed a meta-analysis of 53 studies [48]. In the above investigation, it was reported that the T allele of rs2241766 increases the risk of T2DM in the West Asian population while the same allele reduces the risk of T2DM in the South Asian population.

Ethnic variations in the association of rs2241766 with chronic diseases predisposed by insulin resistance have been reported in previous studies. Wu et al. reported that rs2241766 is associated with an increased risk of obesity in Chinese populations only, lacking such an association in non-Chinese people [49].

The findings of this investigation support that the G allele of rs2241766 reduces the risk of poor glycemic control in T2DM, while the T allele increases its susceptibility. Jordan is a Middle Eastern country. Given the above discussion demonstrating the presence of ethnic variations in the role of rs2247166 in determining the risk of multiple metabolic disorders, it would be interesting to test whether the role of rs2247166 in determining the risk of poor glycemic control is affected by race or ethnicity.

Body fat distribution affects serum adiponectin levels. One limitation of this report was the lack of any measurement that reflects body fat distribution, such as waist-hip [50] or visceral fat ratios [51]. Another limitation was our failure to collect information on the eating behavior of the participants, such as skipping breakfast or eating slowly. These factors were shown in previous reports to affect glycemic control [52].

In conclusion, this report demonstrated that serum adiponectin levels could influence glycemic control in T2DM patients. This effect could be partially explained by genetic variations in the gene that codes for adiponectin.

## Figures and Tables

**Table 1:** *ADIPOQ* SNPs information.

SNP ID	Location and base change	Forward primer reverse primer	PCR program (34 cycles)	PCR amplicon size (bp)	Restriction enzyme, incubation temperature, and time	RFLP product (BP)
rs17300539	Promoter∗ region (A/G)	CAGGAAGCTGAGGCTTTACA ACCCACTTAGGTGTTCCTAGA	95°C 30 sec, 58.5°C 30 s, 72°C 1 min	342	Alu I 37°C, 1 h	AA: 236, 106 AG: 342, 236, 106 GG: 342

rs266729	Promoter∗ region (C/G)	ACTGTGGAGATGATATCTGG CATTTTGACAGCTACCTTGG	95°C 30 sec, 58.5°C 30 s, 72°C 1 min	412	Hha1 37°C, 1 h	GG: 170, 243 CG: 170, 243, 412 CC: 412

rs1501299	Intron 2∗ (G/T)	TGACCAGGAAACCACGACTC CCATCTACACTCATCCTTGG	95°C 30 sec, 58°C 30 s, 72°C 1 min	341	BsmI 65°C, 1 h	GG: 229, 112 GT: 341, 229, 112 TT: 341

rs2241766	Exon 2∗ (T/G)	AGTAGACTCTGCTGAGATGG ACATTCTTACCTGGATCTCC	95°C 30 sec, 59°C 30 s, 72°C 1 min	333	BspH1 37°C, 1 h	TT: 153, 180 TG: 333, 153, 180 GG: 333

Abbreviations: SNP: single nucleotide polymorphism; PCR: polymerase chain reaction; RFLP: restriction fragment length polymorphism. All SNP information was obtained from the NCBI dbSNP database.

**Table 2:** Baseline variables of the study subjects.

Variable	Good glycemic control *n* = 175	Poor glycemic control *n* = 175	*P* value
Age (years)	58.77 ± 9.86	58.89 ± 9.99	0.9057
Gender (*n*) (%)			0.0645
Male	63 (36%)	80 (45.7%)	
Female	112 (64%)	95 (54.3%)	
BMI (kg/m^2^)	31.37 ± 5.72	31.14 ± 5.57	0.7128
WC (cm)	106.47 ± 9.80	107.92 ± 12.85	0.2357
Cholesterol (mg/dl)	202.57 ± 59.55	203.32 ± 62.22	0.9072
Triglyceride (mg/dl)	153.91 ± 93.61	171.68 ± 106.89	0.0998
Treatment duration (years)	4.90 ± 5.29	8.41 ± 6.07	<0.0001
HbA1c	6.16 ± 0.50	8.91 ± 1.59	<0.0001
Glucose (mg/dl)	167.71 ± 68.92	239.44 ± 105.18	<0.0001
HOMA-IR	1.91 ± 1.97	4.04 ± 4.99	<0.0001
Adiponectin (*μ*g/ml)	9.56 ± 16.45	4.80 ± 7.47	<0.0006

Abbreviations: BMI: body mass index; WC: waist circumference; HbA1c: glycated hemoglobin; HOMA-IR: homeostatic model assessment insulin resistance. Data are presented as mean ± standard deviation. The *P* values were calculated by Student's *t*-test except for gender distribution which was calculated using Pearson's chi-square.

**Table 3:** Multivariate regression analysis of the study subjects.

Variable	OR	95% CI	*P* value
Adiponectin	0.974	0.949-0.999	0.043
Treatment duration	1.114	1.064-1.166	0.0001
Cholesterol	1.000	0.994-1.004	0.915
Triglycerides	1.000	1.000-1.000	0.965
HOMA-IR	1.584	1.331-1.886	0.0001
Constant	0.181	—	0.001

Abbreviations: OR: odds ratio; CI: confidence interval; HOMA-IR: homeostasis model assessment-insulin resistance.

**Table 4:** Genotype frequencies of various ADIPOQ SNPs in good and poor glycemic control subjects.

SNP ID	Genotype	Good glycemic control (*n* = 175)	Poor glycemic control (*n* = 175)	*P* value
rs17300539	GG	148 (84.5%)	160 (91.4%)	0.0890
	GA	26 (15.0%)	15 (8.6%)	
	AA	1 (0.5%)	0 (0.0%)	

rs266729	CC	112 (64.0%)	107 (61.0%)	0.3500
	CG	59 (33.7%)	59 (33.7%)	
	GG	4 (2.3%)	9 (0.05%)	

rs2241766	TT	54 (30.9%)	100 (57.1%)	<0.0001
	TG	109 (62.3%)	68 (38.9%)	
	GG	12 (6.8%)	7 (4.0%)	

rs1501299	GG	77 (44.0%)	79 (45.2%)	0.9500
	GT	73 (41.7%)	73 (41.7%)	
	TT	25 (14.3%)	23 (13.1%)	

The *P* values were calculated using Pearson's chi-square test of association.

**Table 5:** Allele frequencies of various ADIPOQ SNPs in good and poor glycemic control of T2DM subjects.

SNP ID	Allele	Good glycemic control *n* (%)	Poor glycemic control *n* (%)	*P* value
rs17300539	G	322 (92.0%)	335 (96.0%)	0.0265
	A	28 (8.0%)	14 (4.0%)	

rs266729	C	283 (81.0%)	273 (78.0%)	0.3497
	G	67 (19.0%)	77 (22.0%)	

rs2241766	T	217 (62.0%)	268 (77.0%)	0.0001
	G	133 (38.0%)	82 (23.0%)	

rs1501299	G	227 (65.0%)	231 (66.0%)	0.7505
	T	123 (35.0%)	119 (34.0%)	

The *P* values were calculated using Pearson's chi-square test of association.

**Table 6:** Haplotype frequencies of SNPs rs17300539, rs266729, rs2241766, and rs1501299 in good glycemic control and poor glycemic control.

Haplotype	rs17300539	rs266729	rs2241766	rs1501299	Good glycemic control frequency	Poor glycemic control frequency	OR (95% CI^2^)	*P* value
1	A	C	T	T	0.029	0.039	1.276 (0.553-2.901)	0.5751
2	G	C	G	G	0.244	0.153	0.500 (0.341-0.734)	0.0003
3	G	C	G	T	0.052	0.065	1.152 (0.610-2.175)	0.6627
4	G	C	T	G	0.241	0.326	1.362 (0.974-1.905)	0.0704
5	G	C	T	T	0.202	0.195	0.861 (0.592-1.252)	0.4331
6	G	G	T	G	0.117	0.165	1.351 (0.876-2.083)	0.1722
7	G	G	T	T	0.019	0.040	1.991 (0.776–5.108)	0.1449

Data were automatically generated by the SHEsis software. Abbreviations: OR: odds ratio; CI: confidence interval. The *P* values were calculated using Pearson's chi-square test of association.

## Data Availability

The datasets generated and/or analyzed during the current study are available from the corresponding author on reasonable request.
